# Electronic monitoring of doffing using video surveillance to minimise error rate and increase safety at Howard Springs International Quarantine Facility

**DOI:** 10.1186/s13756-022-01155-2

**Published:** 2022-09-30

**Authors:** Stephanie J. Curtis, Abigail Trewin, Kathleen McDermott, Karen Were, Kate Clezy, Kathy Dempsey, Nick Walsh

**Affiliations:** 1 National Critical Care and Trauma Response Centre, 5 Lancaster Rd, Eaton, NT Australia; 2 Clinical Excellence Commission, 1 Reserve Rd, St Leonards, NSW Australia

**Keywords:** Personal protective equipment, Hand hygiene, Safety management, Compliance, Observation, Disease outbreaks, COVID-19, Quarantine, Emergencies, Epidemiology

## Abstract

**Background:**

Safe donning and doffing of personal protective equipment (PPE) are critical to prevent transmission of infectious diseases. Novel strategies to improve infection prevention and control (IPC) adherence can optimise safety. We describe and quantify video surveillance of doffing at an outdoor hotel quarantine facility led by the Australian Medical Assistance Team in the Northern Territory, Australia.

**Methods:**

Motion-activated video cameras were installed in seven areas where personnel doffed PPE upon exit from an area dedicated to quarantined residents. Video footage was reviewed daily and compliance issues were identified using a standardised checklist and risk graded to initiate feedback. We collated audit data from 1 February to 18 April 2021 to describe trends by month, staff group, doffing component and risk.

**Results:**

In 235 h of video footage, 364 compliance issues were identified, of which none were considered high-risk compromising to PPE integrity. Compliance issues were low risk (55/364, 15%) or moderate risk (309/364, 85%) and the most common issue was missed or inadequate hand hygiene (156/364, 43%). Compliance issues per minute of video footage reviewed decreased following introduction of the activity, from 24 per 1000 in February to 7 per 1000 in March and April.

**Conclusion:**

Video surveillance with feedback supported rapid response to improve IPC adherence in a challenging ambient environment. The activity focused on perfection to identify compliance issues that would go unreported in most healthcare settings and contributed to a suit of activities that prevented any high-risk PPE breaches or compromises to safety.

**Supplementary Information:**

The online version contains supplementary material available at 10.1186/s13756-022-01155-2.

## Introduction

Safe donning and doffing of personal protective equipment (PPE) are critical to protect patients and staff from transmitting infectious diseases. To ensure PPE is donned and doffed in the correct sequence, with appropriate hand hygiene between each step and disposal of equipment, competency-based training and audits with regular and timely performance feedback are recommended [1, 2]. However, poor adherence to infection prevention and control (IPC) procedures is common in healthcare despite an understanding of the importance for safety and as a condition of employment [3]. Therefore, novel monitoring strategies may be required to improve adherence to IPC procedures.

Video surveillance with feedback has successfully improved hand hygiene, donning and doffing in the hospital setting [4–8]. Video surveillance is a form of direct observation that can lead to the Hawthorne effect, i.e., the modification of an individual’s behaviour when being observed, and can reduce psychological stressors of working in a high-risk environment, as it builds employee confidence that risk is adequately managed [8, 9]. Despite these benefits, the use of video surveillance has been limited to research projects and, to a lesser extent, hospital settings. These settings have also tended to concentrate on video reflexive ethnography for teaching opportunities rather than surveillance [10]. We describe and quantify the use of video surveillance with feedback of PPE doffing performed as part of operations in an outdoor hotel quarantine facility in Australia.

## Methods

### Setting and study population

Howard Springs International Quarantine Facility at the Centre for National Resilience was the Australian Medical Assistance Team (AUSMAT) operation responsible for the repatriation of Australian citizens and permanent residents unable to return to Australia by commercial flights from 23 October 2020 to 23 May 2021 [11]. Over this period, the centre quarantined 7105 residents, including 205 confirmed COVID-19 cases, and there was no leakage of severe acute respiratory syndrome coronavirus 2 (SARS-CoV-2) from residents to staff or the community, with Alpha and Delta the dominant variants of concern identified in SARS-CoV-2 infected residents. The operation was in the Northern Territory, which has a harsh tropical savanna climate with high humidity and temperature [12]. AUSMAT led the operation through partnership with local contractors (catering, concierge, high cleaners, deep cleaners, maintenance and waste management), local and federal police, and the Australian Defence Force.

### Intervention

In mid-January 2021, following an increased number of residents accepted to quarantine at the facility, motion activated video cameras were installed at five doffing stations. The video surveillance had been successfully piloted at two doffing stations for the previous month. In March, two additional doffing stations were established and video monitored. All personnel were informed of the monitoring daily, and entry signage of video monitoring in progress was advised. The cameras were positioned to view the whole doffing station to record personnel doffing PPE upon exit from an area dedicated to quarantined residents. Personnel had donned PPE required for activities considered either low or high risk. High-risk activities was classified as face-to-face resident contact or terminal room cleaning, which required PPE for airborne precautions, including P2/N95 mask, eyewear, face shield, gloves and gown. All other activities were low risk and required gloves, surgical masks and eyewear. All personnel operated in a buddy system for all activities at the quarantine facility.

Video footage was collected from each camera’s Secure Digital card and reviewed daily by a trained security officer through a compliance checklist developed by AUSMAT, based on the World Health Organization minimum guidelines (Additional file 1) [13]. When personnel’s doffing did not meet all checklist criteria, if the procedure was performed in the incorrect order, or if personnel was wearing jewellery that was not identified during donning, the activity was considered a potential compliance issue. Potential compliance issues were reported daily to the AUSMAT clinical leadership team, who confirmed if the issue was reportable and provided a risk grading. The leadership team completed an additional weekly review of sampled videos and audit sheets as a quality assurance measure. There was a high index of error-finding with a goal of perfection, as defined in Table 1.

**Table 1:** Risk grading and follow-up action of reportable compliance issues detected through doffing video surveillance

Risk grading	Definition	Component of compliance issue	Follow-up action
Green	Minor errors in technique that created no additional risk or compromise to PPE integrity but did not fulfil procedural perfection	Hand hygiene: not performed between each step of the doffing sequence once or twice, or not performed for the required duration (20 s) up to twice between each step of the doffing sequence PPE order: incorrect doffing sequence or incorrect method of removal for one step Mask, glasses, gown, gloves and face shield: standing too close (< 1 m) to the infectious waste bin during doffing Jewellery: wearing of earrings	Analysis of trends and feedback through daily group-level hand hygiene and PPE training
Blue	Moderate errors in technique that created some risk but did not compromise PPE integrity	Hand hygiene: not performed between each step of the doffing sequence three or more times, or not performed for the required duration (20 s) three or more times between each step of the doffing sequence PPE order: incorrect doffing sequence or incorrect method of removal for two steps Mask, glasses, gown, gloves and face shield: PPE contact with the bin before completing the doffing sequence, glasses not cleaned, touching the front of the mask or the mask/face shield travelling up during doffing Jewellery: wearing of a watch, bracelet, necklace or lanyard	Individual consultation (within 24-h) for retraining, demonstration of the correct procedure and ongoing individual monitoring for improvements
Red	Major error with compromise to PPE integrity	Hand hygiene: not performed or not performed at the conclusion of doffing PPE order: incorrect doffing sequence for three or more steps or a catastrophic failure of PPE removal resulting in compromise to clothing, skin or membranes Mask, glasses, gown, gloves and face shield: failure to use required PPE, use of unapproved equipment additions or mask travelling over face during removal Jewellery: not applicable to red risk grading	Immediate individual consultation (via phone call or face-to-face, whichever was quicker) with retraining, demonstration of the correct procedure and investigation to determine isolation requirements

Following risk grading, the AUSMAT clinical leadership team initiated the follow-up action required (Table 1). Subsequently, data were aggregated according to staff group and by component (hand hygiene, face shield, gloves, gown, glasses, mask, jewellery, or sequence order). In addition to this surveillance, individual PPE breach reporting were mandatory, and reporting of near misses and potential compliance issues experienced or witnessed were encouraged but were not included in the video surveillance reporting. Further detail on the intervention are provided in additional file 2.

### Analysis

We collated operational audit data stored in Microsoft Excel from 1 February to 18 April 2021 to describe trends by month, staff group, component of compliance issue and risk. To compare the number of each staff group monitored, we used paper security logs which were mandatory to complete for each entrance to the resident zone (Additional file 3).

## Results

From 1 February to 18 April 2021, 14,084 min of video footage were audited from 38,323 min captured. There were 364 reportable compliance issues identified; 85% (309/364) were moderate risk, 15% (55/364) were low risk and none were high risk. Compliance issues per minutes of video footage reviewed decreased from 24 per 1000 in February to 7 per 1000 in March and April (Table 2).

**Table 2:** Doffing video surveillance results from 1 February to 18 April 2021, by month and overall

	Month			Total, N
	February, N	March, N	April, N	
Residents with COVID-19	25	13	40	78
Total residents	1382	1381	721	3484
Resident prevalence of COVID-19, %	1.8%	0.9%	5.7%	2.2%
Footage captured (minutes)	6337	16,531	15,455	38,323
Footage reviewed (minutes)	2026	6083	5975	14,084
Footage reviewed, %	32.0%	36.8%	38.7%	36.8%
Potential compliance issues identified	229	165	177	571
Reportable compliance issues	153	109	102	364
Potential compliance confirmed to be reportable, %	66.8%	66.1%	57.6%	63.8%
Compliance issues per minutes of footage reviewed	24	7	7	9
*Staff group of reportable compliance issues*				
Cleaning contractors	135	108	28	271
Waste management contractors	4	0	0	4
Catering contractors	0	0	2	2
Other contractors	5	0	42	47
AUSMAT clinical and operations	6	0	16	22
Defence/police	3	1	14	18
*Estimate daily number of staff entrances to the resident zone*				
Cleaning contractors	75	44	61	60
Waste management contractors	8	12	6	9
Catering contractors	20	8	17	15
Other contractors	84	60	22	55
AUSMAT clinical and operations	78	91	87	85
Defence/police	51	34	62	49

**Fig. 1:**
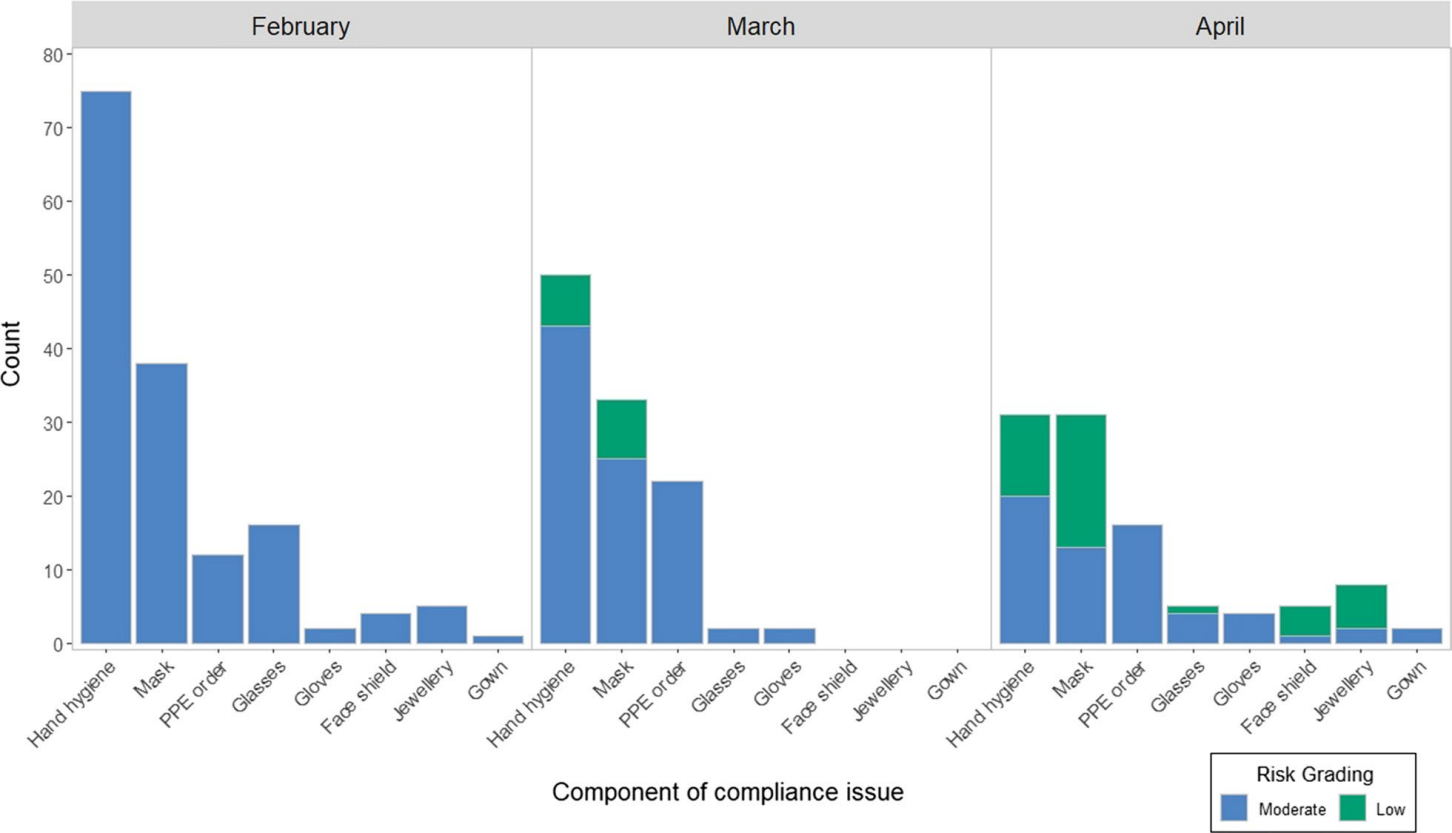
Reportable compliance issues from 1 February to 18 April 2021, by month, component and risk

Cleaning contractors had the highest number of compliance issues reported (271/364, 74.5%) and accounted for 88% (135/153) of compliance issues reported in February. Despite an overall decrease in compliance issues over time, the proportion of compliance issues per staff group decreased for the cleaning contractors to 28% (28/102) in April and increased for all other staff groups except waste management contractors. The most frequent compliance issues were hand hygiene not performed between each step of the doffing sequence or for the required 20-s duration (156/364, 42.9%), mask removal (travelling upwards, touching the front or contact with the infectious waste bin) (102/364, 28.0%) and incorrect doffing sequence or incorrect method of PPE removal for one step (50/364, 13.7%). Compliance issues became increasingly low risk (Fig. 1).

## Discussion

We demonstrated high performance of personnel from various professions to avoid any high-risk PPE doffing breaches in 235 h of video footage reviewed in an outdoor hotel quarantine facility with compounding hazards. Following introduction of the audit activity, compliance issues decreased between February and March, and remained low in April. The compliance issues identified were low or moderate, which would go unnoticed in most healthcare settings but contributed to the avoidance of any high-risk PPE breaches or true compromises to safety.

We report a strong adherence to PPE doffing procedures, which may be explained by the operations prioritisation of creating a safety culture [11]. The hypervigilance of technique and timely feedback of the video surveillance aimed to build team confidence in the level of IPC adherence [14]. Additionally, all staff groups completed daily interactive hand hygiene and PPE training together to perfect technique, build muscle memory and maximise technique during fatigue, considering the rotation of new staff to site this was an excellent risk management tool [15, 16].

An evaluation of the opportunities reviewed indicated PPE doffing compliance issues were initially by contract cleaners, however this increased for other staff groups, predominately other contractors, in April, this may be explained by the higher incident of contractors on site and increased tempo at the facility during this period. There is mixed evidence comparing PPE procedural adherence of non-clinical and clinical staff, however most evidence supports that clinical staff performed better [17–20]. Notably, contract cleaners were the only staff group that did not fall under AUSMAT authority for skill maintenance, leadership or team activities until early April. This change in authority, combined with targeted individual and group training, may explain technique improvement and the reduction in compliance issues identified.

We report the most common compliance issue identified related to missed or inadequate hand hygiene, followed by mask removal. Existing literature reports most doffing procedural errors relate to insufficient hand hygiene, contact with contaminated surfaces or gown removal technique [21–24]. However, existing literature more commonly report ‘breaches’ rather than compliance errors, and our threshold for reporting compliance errors was more sensitive than World Health Organization recommendations, with a goal of perfection for all doffing procedures [13].

Our study has some limitations. Firstly, only one third of all video footage was reviewed, however, in emergency operations 11 weeks is a substantial period, which produced a large amount of performance data for immediate action. Secondly, we were unable to present total doffing events monitored, type of PPE doffed per event or PPE doffing events per positive resident, although we note daily staff entrances to the resident zone and disease prevalence as a proxy. Finally, the use of dedicated staff to perform ongoing surveillance may not be feasible in all settings, however the activity required inexpensive infrastructure and preserved clinical resources through use of a security officer trained to use a structured checklist.

## Conclusion

Video surveillance with feedback supported rapid response to improve IPC adherence and contributed to the prevention of SARS-CoV-2 transmission at the AUSMAT-led quarantine facility. The implementation of novel mitigation strategies should be considered to improve IPC adherence in high-risk settings.

## Supplementary Information

**Additional file 1.** The compliance checklist.

**Additional file 2.** Detail on the intervention.

**Additional file 3.** Detail on methods used to estimate the daily number of staff entrances to the resident zone.

## Data Availability

Data are available from the corresponding author on reasonable request, and subject to permission.

## Abbreviations

AUSMAT: Australian Medical Assistance Team
COVID-19: Coronavirus disease
IPC: Infection prevention and control
PPE: Personal protective equipment
SARS-CoV-2: Severe acute respiratory syndrome coronavirus 2
