# Supplementary material for: Electronic monitoring of doffing using video surveillance to minimise error rate and increase safety at Howard Springs International Quarantine Facility
Source: Antimicrob Resist Infect Control. 2022 Sep 30;11:120. doi: 10.1186/s13756-022-01155-2 (PMC9522442; doi:10.1186/s13756-022-01155-2)
Supplement: Supplementary file 1 — Additional file 1. The compliance checklist. [file 13756_2022_1155_MOESM1_ESM.docx]

**Additional file 1. Australian Medical Assistance Team Personal protective equipment compliance checklist used for doffing video surveillance
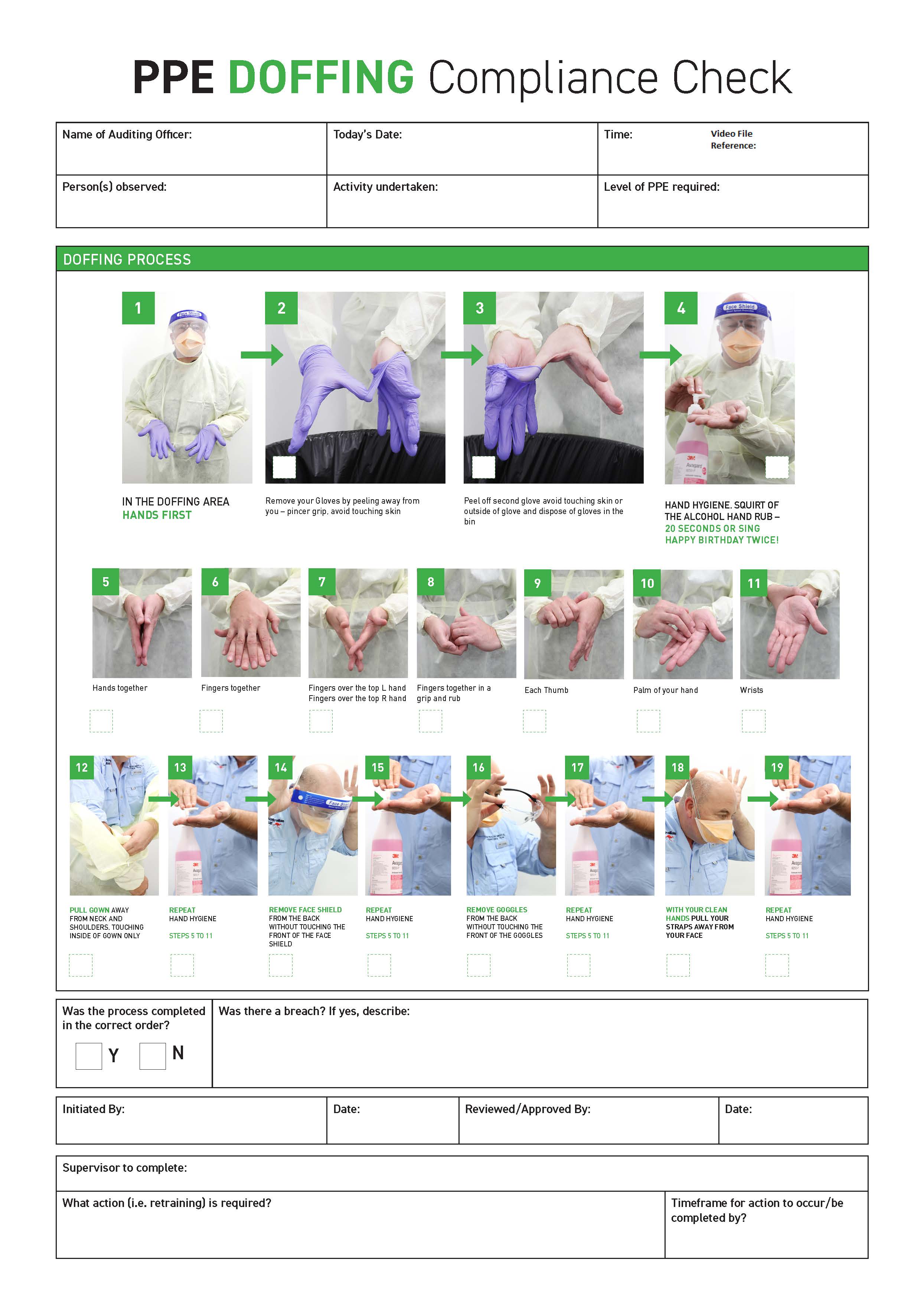
**
