# Supplementary material for: Electronic monitoring of doffing using video surveillance to minimise error rate and increase safety at Howard Springs International Quarantine Facility
Source: Antimicrob Resist Infect Control. 2022 Sep 30;11:120. doi: 10.1186/s13756-022-01155-2 (PMC9522442; doi:10.1186/s13756-022-01155-2)
Supplement: Supplementary file 2 — Additional file 2. Detail on the intervention. [file 13756_2022_1155_MOESM2_ESM.docx]

**Additional file 2. Further operational detail on doffing video surveillance at Howard Springs International Quarantine Facility**

- **Video camera location:** As this was an outdoor quarantine operation, each doffing station was set up in a unique location. Within each doffing station, cameras were positioned to maximise vision of doffing including the bin and sanitation station. Video cameras angles were adjusted to ensure equal vision at each doffing station through review of videos in the first week of operation.
- **Video sampling strategy**: Video review sampling aimed to allow for compliance issue identification across all staff groups whilst considering time availability of the video reviewer. Video recording occurred on a 24-hour basis, however most entries and exits (i.e., donning and doffing performed) within the quarantine areas occurred between 0600 and 2000 daily. The video reviewer prioritised high tempo periods of significant risk – arrival days, high-risk zone entry (confirmed COVID-19 cases and close contacts), to which all rostered personnel were engaged with multiple entries and exits. The high tempo period was usually between 1100 and 1800 over a four-day period each fortnight when a flight cohort was cleared from quarantine, and a new flight cohort commenced quarantine. Contract cleaners predominately entered the facility during high tempo periods, therefore most video footage of their doffing would have been reviewed. Outside of these high tempo periods, the video reviewer would complete as many additional hours as possible of footage for the week, using footage from the Secure Digital (SD) cards which were uploaded to a hard drive by date. The use of motion-activated video cameras decreased the total hours of video surveillance as it did not record when personnel were not actively moving (i.e., were not performing doffing).
- **Security officer training**: The security officer undertook a week of supervised training under the infection prevention control lead and had participated as a “spotter” (an in-person supervisor of doffers) for the two months prior to installation of the video surveillance equipment. The security officer’s accuracy, understanding and direction over several weeks was evaluated by senior infection prevention control staff in the immediate period prior to engagement in the video surveillance role, and weekly random audits were conducted on the video audit process for quality assurance and consistency of performance across the team.
- **Inter-rater reliability:** As this was an operational activity, no formal inter-rater reliability statistical analysis was performed. However, we optimized inter-rater reliability through use of a standardised audit checklist for the activity and through AUSMAT clinical leadership reviewing all potential compliance issues reported, confirming if the issue was reportable and providing a risk grading.
- **Video reviewer fatigue:** The video reviewer completed a four-hour shift Monday-Friday and a ten-hour shift on Saturday and Sunday. The video reviewer was seated in an airconditioned unit with regular breaks. Three AUSMAT support officers that reviewed videos in the initial pilot period over December-January, collected the SD cards daily and uploaded the video recordings to the hard drive by date for the video reviewer.
- **Overview of each doffing station:**
  - **Stations 1 and 2:** used for arrival days by a high volume of staff across the following staff groups: contractor cleaners, contract high-cleaners, catering contractors, AUSMAT clinical and operations, defence and police personnel. These doffing stations were also used throughout the entire day by AUSMAT clinical and operations, defence and police personnel and in the evening by catering contractors.
  - **Stations 3 and 4:** used daily with medium volume throughout the entire day by all staff groups.
  - **Station 5:** used daily with low-volume throughout the entire day by a small number of personnel entering the high-risk zone, including AUSMAT clinical and operations and contract high cleaners.
- **Feedback to staff:** All reportable compliance issues identified were raised at the daily all staff interactive hand hygiene and PPE training, with an emphasis on how and why to minimise errors relating to the recently identified compliance issues. Training included simulating 20 seconds of hand hygiene at every step in the doffing process and asking staff to count in unison to achieve the correct time. Contract cleaners were the only staff group that did not fall under AUSMAT authority for skill maintenance, leadership or team activities until early April. Prior to this, all reportable compliance issues relating to this cohort were directly raised by the Mission Lead to their supervisors alongside offers of assistance to train staff. Furthermore, additional infection prevention and control staff attended the doffing stations at the conclusion of cleaning periods to assist with spotting and minimise compliance issues.
