# Supplementary material for: Electronic monitoring of doffing using video surveillance to minimise error rate and increase safety at Howard Springs International Quarantine Facility
Source: Antimicrob Resist Infect Control. 2022 Sep 30;11:120. doi: 10.1186/s13756-022-01155-2 (PMC9522442; doi:10.1186/s13756-022-01155-2)
Supplement: Supplementary file 3 — Additional file 3. Detail on methods used to estimate the daily number of staff entrances to the resident zone. [file 13756_2022_1155_MOESM3_ESM.docx]

**Additional file 3. Method used to estimate the daily number of staff entrances to the resident zone**

We categorised all dates over the audit period into three groups; (1) flight arrival day of new quarantine residents, (2) flight departure day of residents following completion of 14-days quarantine (excluding confirmed COVID-19 cases that had their quarantine period extended), or (3) non-flight arrival or departure day. We randomly selected one date for each of these categories per month and reviewed all paper logs to quantify the amount of each staff group entrances to the resident zone for the respective day. Paper logs were maintained by security officers that were stationed at each quarantine area point of entry to maintain mandatory records of all personnel entries, including name, time, employer and purpose of entry. For each category of day per month and staff group, we multiplied the total staff entrances by the total number of days in the month relevant to that category and summed these numbers together. Subsequently, we divided the summed result by the total number of days that the video surveillance was performed to account for the differences in follow-up days per month. Finally, we summed the estimates for the three categorised days to estimate the daily number of staff entrances to the resident zone. Table 1 presents the parameters, and an example equation to estimate the daily number of AUSMAT staff entrances to the resident zone in February is as followed: $\frac{(\left( 98\times8 \right)+\left( 102\times6 \right)+\left( 56\times14 \right))}{28}=78$

**Table 1. Parameters used to estimate the daily number of each staff group entering the resident zone.**

|  | **February** | **March** | **April** |
| --- | --- | --- | --- |
| Total days in video surveillance | 28 | 31 | 18 |
| Total flight arrival days | 8 | 6 | 4 |
| Total flight departure days | 6 | 9 | 4 |
| Total non-flight arrival or departure days | 14 | 14 | 10 |
| **Total entries on flight arrival day** | | | |
| Cleaning contractors | 0 | 0 | 190 |
| Waste management contractors | 10 | 13 | 10 |
| Catering contractors | 15 | 0 | 22 |
| Other contractors | 146 | 167 | 21 |
| AUSMAT clinical and operations | 98 | 90 | 49 |
| Defence/police | 48 | 37 | 37 |
| **Total entries on flight departure day** | | | |
| Cleaning contractors | 168 | 67 | 40 |
| Waste management contractors | 6 | 11 | 10 |
| Catering contractors | 10 | 11 | 20 |
| Other contractors | 88 | 40 | 35 |
| AUSMAT clinical and operations | 102 | 121 | 147 |
| Defence/police | 34 | 71 | 59 |
| **Total entries on non-flight arrival or departure days** |  |  |  |
| Cleaning contractors | 77 | 54 | 18 |
| Waste management contractors | 8 | 13 | 3 |
| Catering contractors | 27 | 11 | 14 |
| Other contractors | 47 | 36 | 17 |
| AUSMAT clinical and operations | 56 | 85 | 79 |
| Defence/police | 60 | 13 | 73 |
| **Estimate daily number of staff entrances to the resident zone** | | | |
| Cleaning contractors | 75 | 44 | 61 |
| Waste management contractors | 8 | 12 | 6 |
| Catering contractors | 20 | 8 | 17 |
| Other contractors | 84 | 60 | 22 |
| AUSMAT clinical and operations | 78 | 91 | 87 |
| Defence/police | 51 | 34 | 62 |
